# Impact of Exercise Training on Depressive Symptoms in Cancer Patients: A Critical Analysis

**DOI:** 10.3390/biology11040614

**Published:** 2022-04-18

**Authors:** Priscila Marconcin, Adilson Marques, Gerson Ferrari, Élvio R. Gouveia, Miguel Peralta, Andreas Ihle

**Affiliations:** 1 Centro Interdisciplinar de Estudo da Performance Humana, Faculdade de Motricidade Humana, Universidade de Lisboa, 1495-751 Lisbon, Portugal; amarques@fmh.ulisboa.pt (A.M.); mperalta@fmh.ulisboa.pt (M.P.); 2 KinesioLab, Research Unit in Human Movement Analysis, Piaget Institute, 2805-059 Almada, Portugal; 3 ISAMB, Universidade de Lisboa, 1649-020 Lisbon, Portugal; 4 Laboratorio de Rendimiento Humano, Grupo de Estudio en Educación, Actividad Física y Salud (GEEAFyS), Universidad Católica del Maule, Talca 3460000, Chile; gerson.demoraes@usach.cl; 5 Department of Physical Education and Sport, University of Madeira, 9020-105 Funchal, Portugal; erubiog@uma.pt; 6 Laboratory of Robotics and Systems in Engineering and Science (LARSyS), Interactive Technologies Institute, 9020-105 Funchal, Portugal; 7 Center for the Interdisciplinary Study of Gerontology and Vulnerability, University of Geneva, 1205 Geneva, Switzerland; andreas.ihle@unige.ch; 8 Department of Psychology, University of Geneva, 1205 Geneva, Switzerland; 9 Swiss National Centre of Competence in Research LIVES—Overcoming Vulnerability: Life Course Perspectives, 1015 Lausanne, Switzerland

**Keywords:** tumour, exercise, depression, mental health, cancer survivorship

## Abstract

**Simple Summary:**

Cancer patients need to overcome several issues, leaving them more vulnerable to depressive symptoms. Exercise is recognised as a practice that helps to deal with depressive symptoms. This study is an umbrella review of meta-analyses about the effect of exercise on depressive symptoms among cancer patients. Six studies were included. A significant reduction in depressive symptoms was observed because of exercise. However, the studies varied in methodological terms, making a broad generalisation difficult. We can conclude that exercise is a good alternative to deal with depressive symptoms among cancer patients. Still, more studies are needed to clarify some aspects that are not answered yet.

**Abstract:**

Background: Cancer patients must deal with several health challenges, including emotional distress and depressive symptoms. This study aimed to evaluate evidence from published systematic reviews and meta-analyses about the efficacy of exercise on depressive symptoms in cancer patients. Methods: We searched for previous meta-analyses of randomised controlled trials on PubMed, Web of Science and Scopus, with data inception to 30 December 2021. Two independent researchers assessed the methodological quality using the Assessment of Multiple Systematic Reviews 2 (AMSTAR2) instrument. Six meta-analyses were integrated. All included middle-aged and older adults. Five presented moderate quality, and one presented low quality. Results: Overall, a significant reduction in depressive symptoms was observed among the included studies. However, the heterogeneity between studies was high, and high-quality evidence for the efficacy of exercise on depressive symptoms was limited. Conclusions: Exercise could be a possibility in the treatment of depressive symptoms in cancer patients, especially when supervised and outside the home. The better dose of exercise needs to be clarified. More high-quality evidence is needed to better prescribe exercise to this vulnerable population.

## 1. Introduction

Cancer is a global public health issue, with 19.3 million new cases of cancer diagnosed in 2020 and 10 million individuals dying from the disease [1]. Cancer occurs mostly with older age and in the United States of America, and 90% of cancers are diagnosed in those aged >50 years [2]. Female breast cancer is the most commonly diagnosed cancer (11.7%), followed by lung (11.4%), colorectal (10.0%), prostate (7.3%), and stomach (5.6%) cancers [1]. Despite the lethality of different types of cancer, many cancer patients survive. However, cancer patients are in a vulnerable situation since they go through several health challenges, as cancer diagnosis and treatment have a serious impact on their physical and mental well-being [3]. Cancer patients experience several emotional disruptions, such as fear of death, interruption of life plans, decreased body image and self-esteem, and changes in social role and lifestyle [4]. One of the most common impacts is depression, which affects up to 20% of patients with cancer [5], however, the prevalence rate of depression among cancer patients is heterogeneous, according to clinical setting [6], the stage of the disease [5,7] and type of cancer [8], ranging between 5% and 49% [9]. Aggravating this issue, depression in cancer patients is associated with low chemotherapy compliance [10] and an increased risk of death [11]. Therefore, the treatment of depression among cancer patients should be a priority. However, there is still the notion that depression is inevitable and untreatable [12]. In addition, there is limited trial data on depressive symptoms’ treatment efficacy in cancer patients [13]. Pharmacological therapy, consisting of antidepressant medications, is usually considered for the treatment of moderate to severe major depression; also, a combined modality approach, including psychosocial and pharmacologic interventions, is a feasible alternative [14].

Alongside pharmacological and psychosocial therapy, exercise can have a positive impact on depressive symptoms [15]. Several mechanisms are involved in the association between exercise and depression, from neurobiological to behavioural mechanisms [16]. One is the inflammation-related factors (IRFs) [17], where studies have shown an association between inflammatory markers and depressive symptoms, including fatigue, impaired sleep and cognitive dysfunction [18,19]. Exercise could create an anti-inflammatory environment and reduce the serum level of leptin and fibroblast growth factors (FGF) [20]. IL-10, produced by exercise, acted as an anti-inflammatory cytokine and is stimulated by the release of adrenaline and cortisol from the adrenal gland, which reduces the release of pro-inflammatory cytokines in the hippocampus [21]. Regarding behavioural mechanisms, exercise can promote several behavioural changes. Engagement in exercise programs and learning new movements skills or completing physically challenging exercises may lead to gaining a sense of mastery [22]. The activity-based perception of physical strength and flexibility is associated with increased physical self-esteem and consequently, an increase in global self-esteem [23].

Regular exercise after diagnosis increases survivorship by 50–60%, with strong evidence for breast and colorectal cancers [15]. In addition to improving depressive symptoms, exercise positively impacts other depression- and cancer-related outcomes, such as anxiety, fatigue, physical functioning, and health-related quality of life [3]. Although the efficacy of exercise interventions in reducing depressive symptoms among cancer patients was already established by previous systematic reviews and meta-analyses [24,25,26,27,28,29], previous studies substantially vary in scope, quality and methodology, which can cause considerable confusion and misdirect efforts in the implementation of exercise interventions. An umbrella review of previous research is warranted to better inform future trials needs, as well as establish a consistent message for health policies targeting this vulnerable population. The specific questions that we should answer with this study are: (1) regarding some aspects of exercise intervention, such as the type of exercise, the dose of exercise, the difference between home-based exercise and other locations, which are the most effective to deal with depressive symptoms? (2) Regarding the difference between the type of cancer, the moment of the exercise intervention, before, during or after cancer treatment, are there any differences? Therefore, this study aimed to present an umbrella review of an exercise intervention on depressive symptoms among cancer patients, appraising hints of uncertainty and bias in the body of literature and providing recommendations for future research.

## 2. Materials and Methods

### 2.1. Literature Search

The protocol of this umbrella review was registered under PROSPERO (CRD42021254843) and followed the Preferred Reporting Items for Systematic reviews and Meta-Analyses (PRISMA) 2020 guidelines [30]. Two researchers performed the literature search in PubMed, Web of Science, and Scopus, focusing on meta-analyses published until 30 December 2021 to investigate the efficacy of exercise in reducing depressive symptoms among cancer patients. In cases of disagreement, a third researcher was asked to arbitrate. The search terms were: (“physical activ*” OR “physical inactiv” OR exercise OR training OR sport* OR fitness OR “movement behavio*” OR walking OR running OR yoga OR jogging OR swimming OR cycl*) AND (depress* OR “mental health” OR mood OR “psychological health” OR “psychological function*” OR “mental function*” OR worries OR worry OR “depressive disorder*” OR “baby blues”) AND (cancer OR neoplas* OR tumor OR chemo* OR radiat* OR malign* OR carciniom*) NOT Rats. No language limitation was established. Records previously known to the authors were also identified.

### 2.2. Eligibility Criteria

Included articles in the systematic review met the PICOS (participants, intervention, comparison, outcome, study design) criteria [31]. The criteria included characteristics of participants (cancer patients); intervention (any type of exercise); comparison: regular care or physical activity; outcome (depressive symptoms diagnosed using a structured clinical interview, screened for probable depression using a validated assessment, or diagnosed according to the judgement of a health professional); study design (meta-analyses of parallel designs, controlled trials. Meta-analyses were excluded if the studies involved animals.

### 2.3. Quality Assessment

Two authors assessed the methodological quality of the included meta-analyses using the Assessment of Multiple Systematic Reviews (AMSTAR 2) checklist. Scores range from 0 to 11, with higher scores indicating greater quality [25]. The AMSTAR checklist involves the dichotomous scoring (0 or 1) of 11 items related to the rigour of systematic reviews and meta-analyses (e.g., comprehensive search strategy, publication bias assessment). AMSTAR scores are graded as high (8–11), medium (4–7), and low (0–3) quality [32]. The authors discussed grading discrepancies and reached a consensus.

### 2.4. Data Extraction

Study characteristics were extracted from full texts, including the number of randomised controlled trials (RCTs) and participants; participants’ characteristics; exercise intervention’s characteristics; comparisons; and outcomes measures. Data on the standardised mean difference (SMD) and heterogeneity (I2 statistic) in meta-analytic comparisons were also extracted. The SMD was classified as trivial (<0.20), small (0.20 to 0.49), medium (0.50 to 0.79) or large (≥0.80) [33]. I2 statistic values were considered to be representative of low (0 to 25%), moderate (25 to 50%), large (50 to 75%) or a very large (>75%) inconsistency [34].

## 3. Results

### 3.1. Literature Search

**Figure 1:**
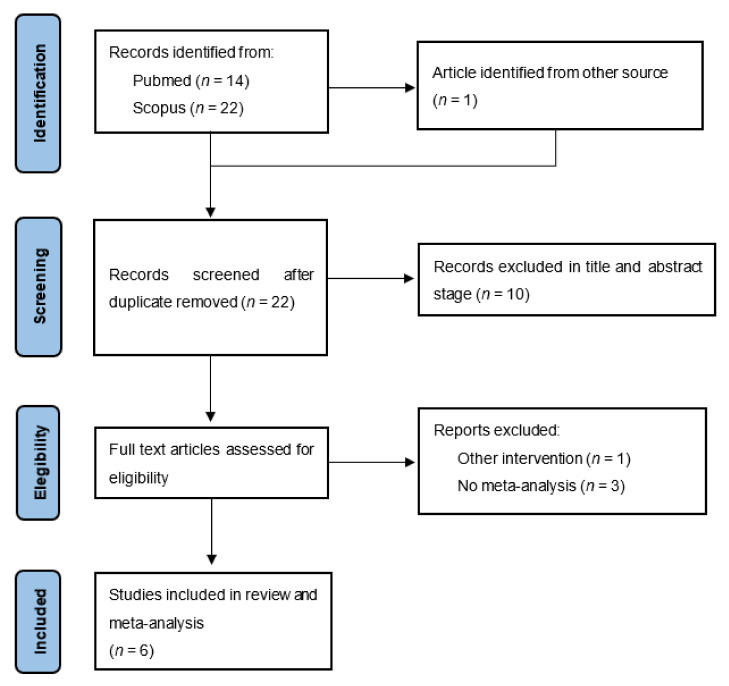
PRISMA flow diagram of study selection.

The study selection process is summarised in Figure 1. A total of 54 records were identified in the literature search, 53 from the databases and 1 from other sources, i.e., previously known to the authors. After removing the duplicates (*n* = 32), two researchers reviewed the remaining 22 records’ titles and abstracts. Ten records were excluded at this stage. The remaining 12 records’ full text were assessed for eligibility. From this analysis, six records were excluded for the following reasons: another type of intervention (*n* = 1); without meta-analysis (*n* = 3); without data on depressive symptoms (*n* = 2). Therefore, six records were included in this study [24,25,26,27,28,29].

### 3.2. Study Characteristics

The characteristics of the meta-analyses included in this umbrella review are presented in Table 1.

### 3.3. Number of RCTs and Participants

The number of participants included in each meta-analysis varied according to the number of included RCTs. The Brown et al. research had the largest sample, including 37 RCTs and 2929 participants [24], while Vashistha et al. presented the smallest sample, with three RCTs and 196 participants [28]. Considering all the included meta-analyses, this study undertook 100 RCTs and 8125 participants. The overlap of single studies within the six included meta-analyses was low (27%), leading to a final number of 79 RCTs.

### 3.4. Participants’ Characteristics

The mean age of the participants ranged between 45 [29] and 73 years [28]. The mean age of five [24,25,26,27,29] out of the six included studies is above 50 years, that is, patients of older age. Two meta-analyses only included women [27,29], the other two meta-analyses included men and women [24,26], and one meta-analysis only included men [28]. One meta-analysis did not present participants’ gender information [25]. Two meta-analyses were focused on breast cancer [27,29]. In three other meta-analyses, most RCTs were focused on breast cancer (24 out of 37 [24], 60% of participants [25], and 18 out of 26 [26]). One meta-analysis was focused on prostate cancer [28].

### 3.5. Exercise Intervention Characteristics

Different types of exercise interventions were included, such as aerobic training (e.g., walking, cycling) [24,25,27,28], resistance training (e.g., weight machines, resistance bands) [24,25,27,28], yoga [24,26,27] and qigong [28]. The duration and session frequency were also different for each intervention. For instance, the mean duration of the intervention in the three meta-analyses that reported this information was 13 weeks [24], 4 to 14 weeks [25], and 9 weeks [26].

### 3.6. Comparison of Experimental Conditions

Exercise interventions were compared with different control conditions, including: no exercise program [24], usual care [25,26,27,28,29], educational print material [25], psychosocial or educational interventions [26], and stretching [27,28].

### 3.7. Outcome Measures

Depressive symptoms were assessed by different instruments, such as the Center for Epidemiological Studies—Depression [24,26,27,28,29], the Profile of Mood States [24,26,29], the Beck Depression Inventory [24,26,29], the Hospital Anxiety Depression Scale [24,26,27,29], the Symptoms Assessment Scale [24], the Patient Health Questionnaire [26], the Brief Symptom Inventory [28], and the Self-rating Depression Scale [29].

### 3.8. Quality Assessment of Studies

All included meta-analyses conducted a risk of bias analysis regarding single studies. Three of them used the PEDro scale. The mean PEDro score was 7.0 ± 1.0 in Brown’s study [24], representing high quality. In the Craft’s study, all with the exception of three studies attained high quality [25]. Additionally, in Patsou’s study, the mean PEDro score indicated high quality (6.1 ± 2.0) [27]. The other three studies used the Cochrane risk of bias tool, assessing six aspects of the trial methodology. Under each domain, studies were classified as low, high or unclear risk of bias. More details about each domain for each study analysed can be seen in the original paper [26,28,29].

### 3.9. Quality Assessment

Table 2 presents the results obtained with the AMSTAR 2 checklist regarding the methodological quality of the meta-analyses. All meta-analyses, except one, presented a moderate-quality review. Vashistha et al. [28] had a low-quality review, mostly because it did not account for the risk of bias in individual studies when interpreting the review results.

### 3.10. Synthesis of Results

#### 3.10.1. Main Results

The main results of each included meta-analysis are summarised in Table 3. Different methods were used to present aggregate effects, including Cohen’s d, Hedges’ g statistic, and the standardised mean difference (SMD) using a random-effects model. In four out of the six included meta-analyses, the authors observed a significant reduction in depressive symptoms favouring the exercise group. In studies from Patsou et al. [20] and Vashistha et al. [21], no statistically significant decrease in depressive symptoms for the exercise group was observed. Three meta-analyses, Brown et al. [17], Craft et al. [18] and Patsou et al. [20] observed small effect sizes, whereas two meta-analyses, Gonzalez et al. [19] and Yi et al. [22], reported moderate effect size. All the included meta-analyses presented a large or very large heterogeneity (I2 from 55% to 84%).

#### 3.10.2. Sensitivity and Subgroup Analyses

Four of the six included meta-analyses presented sensitivity or subgroup analyses. Regarding the type of cancer, the Brown et al. [17] subgroup analysis revealed significant reductions in depressive symptoms among breast cancer patients (d= −0.17; 95% CI: −0.32, −0.02), but non-significant differences for prostate, leukaemia, lymphoma and colorectal cancer patients. Gonzalez et al. [19] proceeded with a subgroup analysis of only breast cancer patients and presented a significant moderate reduction in depressive symptoms favouring exercise (g = −0.41; 95% CI: −0.59, −0.23).

A subgroup analysis compared supervised vs. non-supervised exercise. Brown et al. [17] showed that supervised exercise was the most effective in reducing depressive symptoms (ß = −0.26, *p* = 0.01). Moreover Craft et al. [18] founded that supervised exercise presented a greater reduction in depressive symptoms (ES = −0.67; 95% CI: −1.11, −0.23) than non-supervised exercise (ES = 0.25; 95% CI: −0.01, 0.50).

Another aspect observed in the subgroup analyses was the exercise dose. Craft et al. [18] founded that exercise bout durations >30 min had larger effects (ES = −0.57; 95% CI: −0.91, −0.23) on depression than exercise bouts ≤30 min (ES = 0.01; 95% CI: −0.20, 0.22). Lastly, Patsou et al. [20] demonstrated that exercising ≤135 min/week yielded a moderate to a large effect (g = −0.82; 95% CI: −1.54, −0.10; I2 = 35%) and exercising ≥135 min/week presented no significant effect. Moreover, exercise up to 12 weeks yielded a moderate to a large effect (g = −1.69; 95% CI: −2.66, −0.73; I2 = 32%), while exercise duration over 12 weeks presented no significant effect.

Regarding participants’ age, only Brown et al. [17] explored this subgroup analysis and showed that exercise was the most effective when cancer patients were between 47 and 62 years (ß = −0.27, *p* = 0.01). Among older adults, the effect of exercise on depressive symptoms was not significant.

Craft et al. [18] also analysed potential moderators of effect, including exercise location, observing that home-based exercise was associated with increased depressive symptoms (ES = 0.16; 95% CI: −0.15, 0.47), while other exercise locations presented a reduction in depressive symptoms (ES = −0.45; 95% CI: −0.77, −0.14).

The Gonzalez et al. [19] sensitivity analysis showed that removing one study greatly reduced heterogeneity (I2 = 36.9%) but also reduced the effect size to the small-medium range (g = −0.41; 95% CI: −0.55, −0.28). Additionally, comparing studies that used active and inactive control interventions did not find differences; both had a significant, medium to small effect size post-intervention.

Lastly, Patsou et al. [20] performed several subgroup analyses showing that: aerobic exercise interventions yield a large and significant effect on depression (g = −1.23; 95% CI: −1.97, −0.49; I2 = 0%), no significant effect was found regarding resistance exercise interventions, combined aerobic and resistance exercise and Yoga interventions. Exercise during treatment yielded a moderate effect (g = −0.54; 95% CI: −1.16, 0.08; I2 = 25%), while exercise post-treatment yielded no significant effect.

## 4. Discussion

This umbrella review included six meta-analyses that comprised 100 individual studies with little overlap that investigated the effect of exercise on depressive symptoms among cancer survivors. Overall, a small significant reduction in depressive symptoms in this vulnerable population was observed in the studies. However, high-quality evidence for the efficacy of exercise on depressive symptoms is limited. For a more detailed analysis, some points need to be considered, such as the type of cancer, the specificity of exercise prescription, the time of interventions, and during or after cancer.

In our umbrella review, participants had mainly breast cancer in the included meta-analysis and were mostly women. Only one study did not include breast cancer [27] and was with prostate cancer patients, and it was the one that did not observe a significant effect of exercise on depressive symptoms. In a subgroup analysis, Brown et al. found a significant reduction in depressive symptoms among breast cancer survivors but did not find the same in prostate, leukaemia, lymphoma and colorectal cancer [17]. The prevalence of depression among breast cancer survivors is higher than in other cancers and can achieve 32.8% [35]. Moreover, depression is more prevalent in women than men [36], and breast cancer is prevalent in women. Evidence suggests that depression in breast cancer patients decreases over time and is more common throughout the disease and in the recurrent phase of breast cancer [37]. The occurrence of depression among patients with breast cancer is due to several factors, such as treatment-related distress, worries regarding fear of death and disease recurrence, and altered body image, sexuality and attractiveness [38,39,40]. In addition, a study exposes the association between depression and tumour levels of estrogen receptors and progesterone receptors [41]. A study found that fatigue and pain are significant risk factors for developing depression among breast cancer survivors [37]. Fatigue is also a recognised barrier to exercise [42]; however, exercise can reduce fatigue among women with breast cancer [43]. The benefits of exercise can be extended to improve physical functioning and multiple aspects of quality of life among cancer patients [44]. Moreover, exercise is a feasible alternative to control symptoms burden and improve well-being among breast cancer patients [39].

Another sample characteristic that must be highlighted is that most patients were older adults (>50 years old). In the general population, the prevalence of depression symptoms rises with increasing age, 10% to 15% of older adults have clinically significant depressive symptoms [45]. Older patients with cancer often experience depression, fatigue, pain, and sleep disturbance [46]. Only one included meta-analysis directly explored the role of age in the effectiveness of exercise on depression symptoms and found that the efficacy seems to disappear among old age patients [24]. However, an RCT with older cancer patients receiving chemotherapy found that after the six-week structure exercise program, participants’ anxiety and mood improved [47]. Besides the effects of exercise on mental health, physically active old age patients improve general health, such as physical fitness outcomes, quality of life and increased life expectancy [48].

When considering the effects of exercise on depressive symptoms, it is necessary to consider the characteristics of the exercise we are referring to. Many dimensions of exercise exist, which are captured in part by the principle (frequency, intensity, time and type of exercise), as well as the way of practising, whether accompanied or not and if exercise occurs indoors or outdoors. However, the included systematic meta-analyses showed great variance concerning exercise. Except for Gonzalez et al. and Yi et al. [26,29], which analysed the effects of yoga intervention, the others included meta-analyses that examined a variety of exercises, such as aerobic (e.g., walking, cycling), resistance (e.g., weight machine, resistance bands) and qigong. Only the Patsou et al. study explored the difference between the types of exercise and found that aerobic intervention yields a large significant effect on depressive symptoms. At the same time, resistance training presents a small significant effect, and combined aerobic and resistance training yielded a moderate effect [27]. This statement is in accordance with the American College of Sports Medicine (ACSM), which describes that resistance training alone does not seem effective for depression [3]. Aerobic activities are cost-effective and should be popularised in clinical practice.

Regarding yoga, both included meta-analyses that analysed only intervention found significant and medium effects on depressive symptoms [26,29]. However, in the Patsou et al. study, which included aerobic exercise, resistance exercise and yoga intervention, when a subgroup analysis proceeded and considered only yoga intervention, no significant difference in depression symptoms was observed [27]. The contradictory results found in the three studies can be explained by the fact that yoga combines breathing (pranayama) and meditative techniques during a series of postures (asanas), but different types of yoga were being practised, which made it difficult to understand the effects of this practice [49].

Two included meta-analyses found that supervised exercise is more efficient than non-supervised exercise [32,38], which also appears in the ACSM recommendation [3]. Craft et al.’s study [25] explored the effects of exercise session durations and found that more than 30 min had larger effects compared with less than 30 min of the exercise session. In Patsou et al. [27] ≤135 min/week yielded a moderate to large effect and no effect with ≥135 min/week of exercise. The ACSM describes that aerobic training performed three times per week and for at least 12 weeks or twice weekly with combined aerobic plus resistance training lasting 6 to 12 weeks, can significantly reduce depressive symptoms in cancer survivors during and after treatment. However, the exact exercise duration per week has not yet been established by the ACSM. Gonzalez et al.’s study [26] explored the frequency and found no differences between one class per week and two or more classes per week. In contrast, Patsou et al. [27] explored the exercise intervention program duration and found that exercise for up to 12 weeks yielded a moderate to large effect compared with a small effect of over 12 weeks. Aside from the efficacy of depressive symptoms, exercise has other benefits in health outcomes among cancer survivors that must be considered such as improving cardiorespiratory fitness [50], and muscle strength [51,52].

Another important aspect of the efficacy of exercise on depressive symptoms among cancer survivors is the time of intervention, before the diagnosis, during treatment or in a recovering phase. This aspect was explored, and no difference was found between patients receiving cancer treatment, following treatment or mixed treatment status [26]. On the other hand, patients under treatment yielded a moderate effect, and patients post-treatment yielded a small effect [27]. Exercise increases the chemotherapy completion rate during treatment without causing lymphedema or significant adverse events [53]. In addition, exercise appears to reduce chemotherapy-induced peripheral neuropathy symptoms in patients receiving taxane-, platinum-, or vinca alkaloid-based chemotherapy [54].

Concerning the methodological quality, the AMSTAR 2 scores show that the majority were of moderate methodological quality. Nevertheless, as for item #10, “Did the review authors report on the sources of funding for the studies included in the review”, no study reported the source of funding which can entail a risk of bias.

### Strengths and Limitation

The strength of our study is that we included two recent meta-analyses, one from 2020 [19] and one from 2021 [22], and compiled current data regarding the effectiveness of exercise in depressive symptoms among cancer patients. However, some limitations should be exposed. The prevalence of breast cancer was substantial, which prevented us from generalising the findings to other types of cancer. Two studies did not perform subgroup analysis, and those who performed subgroup analysis did not analyse the same constructs, specifically in relation to the FITT principles of exercise. Future research is required to clarify the effectiveness of different intervention modalities (frequency, intensity, time and type) for depressive symptoms among cancer survivors to allow determination of exercise dose-response. Moreover, additional studies are needed to evaluate the cost-analysis and adverse events of exercise, and there is an urgent need for innovative methods to generate high-quality evidence.

## 5. Conclusions

Our critical review contributed to the evidence of the effects of exercise on depressive symptoms among cancer patients. Four in six studies found a significant effect of exercise. Some aspects should be highlighted and should be used for future interventions. Supervised exercise is better than non-supervised. The dose of exercise seems to be important, however, the finding did not present a specific dose-response relation. Exercise outside of the home is better than home-based exercise. Aerobic exercise is the most effective type of exercise. The effect of exercise on depression seems to be more effective among breast cancer patients. Future studies should explore other types of cancer.

## Figures and Tables

**Table 1:** Characteristics of the meta-analyses included in the study.

Reference	No. of RCTs and Participants	Participants Characteristics	Exercise Intervention’s Characteristics	Comparison	Outcomes Measures
Brown et al. [24]	37 RCTs; 2929 participants.	Age: mean 51.3 years (range: 39–70); Gender: 87% women; Cancer: breast cancer (24 studies), other types of cancer (13 studies).	Type: walking (16 studies), stationary cycling (5 studies), resistance machines (2 studies), resistance bands (3 studies), yoga (8 studies); Duration/frequency: mean of 13.2 ± 11.7 weeks with 3.0 ± 2.5 sessions/week lasting 49.1 ± 27.1 min/session.	Usual care.	Depressive symptoms (CES-D, POMS, BDI, HADS) and Symptom Assessment Scale.
Craft et al. [25]	15 RCTs; 1371 participants.	Age: mean 51.6 years; Gender: no information about gender; Cancer: breast cancer (60% of the included studies).	Type: aerobic (10 studies), aerobic and resistance (5 studies); Duration/frequency: ranged from 4 to 14 weeks; Supervised, facility-based programs (3 studies), unsupervised home-based programs (6 studies), some exercise programs supervised (4 studies).	Usual care (12 studies); Educational print material (3 studies).	Depression inventory and clinician interview.
Gonzalez et al. [26] *	26 RCTs; 1486 participants.	Age: mean 54.4 years (range 44–68.7 years); Gender: 86.1% women; Cancer: breast cancer (18 studies), mixed cancers (2 studies), other types of cancers (6 studies).	Type: hatha yoga (11 studies), other types of yoga (15 studies); Duration/frequency: mean of 9.3, with 1 to 3 sessions/week lasting 45–120 min/session.	Usual care (19 studies); Psychosocial or educational interventions (6 studies); Other physical activity interventions (2 studies).	Depressive symptoms (HADS, BDI-II, CES-D, POMS, PHQ-2, PHQ-9).
Patsou et al. [27]	14 RCTs; 1701 participants.	Age: mean 52 years; Gender: only women; Cancer: only breast cancer.	Type: aerobic, resistance, aerobic and resistance, yoga; Duration/frequency: no information;	Usual care; Health education intervention; Waitlist; Relaxation and stretching.	Depressive symptoms (POMS, HADS, CES-D).
Vashistha et al. [28] **	3 RCTs; 192 participants.	Age: mean between 67 and 73 years; Gender: only men; Cancer: only prostate cancer.	Type: qigong (1 study), aerobic and resistance (1 study), aerobic and light resistance (1 study); Duration/frequency: no information;	Usual care; Stretching.	Depressive symptoms (BSI-18, CES-D).
Yi et al. [29] ***	6 RCTs; 446 participants.	Age: mean between 45 and 60 years; Gender: only women; Cancer: only breast cancer.	Type: only yoga; Duration/frequency: no information;	Usual care.	Depressive symptoms (BDI-II, POMS, HADS, CES-D, SDS).

Abbreviations: BDI, Beck Depression Inventory; BDI-II, Beck Depression Inventory-II; BSI-18, Brief Symptom Inventory; CES-D, Center for Epidemiological Studies—Depression; HADS, Hospital Anxiety Depression Scale; Patient Health Questionnaire-2; PHQ-2, Patient Health Questionnaire-9; PHQ-9; POMS, Profile of Mood States; SDS, Self-rating Depression scale. * Gonzalez et al. [26] analysed 26 RCTs, but 1 was not included in the meta-analysis. ** Vashistha et al. [28] analysed 13 RCTs, but only 3 included measures of depressive symptoms. *** Yi et al. [29] analysed 8 RCTs, but only data regarding the 6 that analysed depressive symptoms is presented.

**Table 2:** Quality of the meta-analyses included in the study according to AMSTAR 2 criteria.

AMSTAR 2 Criteria	Brown et al. [24]	Craft et al. [25]	Gonzalez et al. [26]	Patsou et al. [27]	Vashistha et al. [28]	Yi et al. [29]
1. Did the research questions and inclusion criteria include the components of PICO?	V	V	V	V	V	V
2. Did the review report contain a statement that the review methods were established before the conduct of the review, and did the report justify significant deviations from the protocol?	X	X	V	X	V	X
3. Did the review authors explain their selection of the study designs for inclusion in the review?	V	V	V	V	V	V
4. Did the review authors use a comprehensive literature search strategy?	V	V	V	V	V	V
5. Did the review authors perform study selection in duplicate?	X	V	V	X	V	V
6. Did the review authors perform data extraction in duplicate?	V	V	V	X	V	V
7. Did the review authors provide a list of excluded studies and justify the exclusions?	V	V	V	V	V	V
8. Did the review authors describe the included studies in adequate detail?	V	V	V	V	V	V
9. Did the review authors use a satisfactory technique for assessing the risk of bias (RoB) in individual studies included in the review?	V	V	V	V	V	V
10. Did the review authors report funding sources for the studies included?	X	X	X	X	X	X
11. If meta-analysis was performed, did the review authors use appropriate methods for the statistical combination of results?	V	V	V	V	V	V
12. If meta-analysis was performed, did the review authors assess the potential impact of RoB in individual studies on the results of the meta-analysis or other evidence synthesis?	V	X	V	V	V	V
13. Did the review authors account for RoB in individual studies when interpreting/discussing the review results?	V	V	V	V	X	V
14. Did the review authors provide a satisfactory explanation for, and discussion of, any heterogeneity observed in the review results?	V	V	V	V	V	V
15. If they performed quantitative synthesis, did the review authors carry out an adequate investigation of publication bias (small study bias) and discuss its likely impact on the review results?	V	V	V	V	V	X
16. Did the review authors report any potential sources of conflict of interest, including any funding they received for conducting the review?	V	V	V	V	V	V
	Moderate	Moderate	Moderate	Moderate	Low	Moderate

Note: V when it fulfills the evaluation criterion and X when it does not fulfill the evaluation criterion.

**Table 3:** Results of the meta-analyses included in the study.

Reference	Effect on Depressive Symptoms (95% CI)	I2 (%)	Conclusions
Brown et al. [24]	d = −0.13 (−0.26, −0.01)	55%	Significant small reduction in depressive symptoms compared to usual care among all types of cancer.
Craft et al. [25]	d = −0.22 (−0.43, −0.009)	The test for heterogeneity was significant (*p* < 0.001).	Significant small reduction in depressive symptoms when comparing exercise interventions to control groups.
Gonzalez et al. [26]	g = −0.55 (−0.78, −0.32)	77%	Significant medium effect size in favour of yoga interventions for reducing depression symptoms in comparison to control conditions.
Patsou et al. [27]	g = −0.38 (−0.89, 0.13)	77%	Non-significant reduction in depressive symptoms for the exercise group.
Vashistha et al. [28]	SMD = −3.02 (−7.83, 1.79)	78%	Non-significant reduction in depressive symptoms for the exercise group.
Yi et al. [29]	SMD = −0.56 (−1.05, −0.07)	84%	Significant improvement in depressive symptoms for yoga interventions.

Abbreviations: d, mean change scores (Cohen’s d); g, Hedges’ g statistic to estimated effect size; I2, I-squared statistic for heterogeneity; SMD, standardised mean difference.

## Data Availability

Not applicable.
